# Chicken Cytochrome P450 1A5 Is the Key Enzyme for Metabolizing T-2 Toxin to 3′OH-T-2

**DOI:** 10.3390/ijms140610809

**Published:** 2013-05-23

**Authors:** Shufeng Shang, Jun Jiang, Yiqun Deng

**Affiliations:** Guangdong Provincial Key Laboratory of Protein Function and Regulation in Agricultural Organisms, College of Life Sciences, South China Agricultural University, Guangzhou 510642, Guangdong, China; E-Mails: shangshf101291@163.com (S.S.); jiangjun@scau.edu.cn (J.J.)

**Keywords:** T-2 toxin, chicken CYP1A5, metabolism

## Abstract

The transmission of T-2 toxin and its metabolites into the edible tissues of poultry has potential effects on human health. We report that T-2 toxin significantly induces CYP1A4 and CYP1A5 expression in chicken embryonic hepatocyte cells. The enzyme activity assays of CYP1A4 and CYP1A5 heterologously expressed in HeLa cells indicate that only CYP1A5 metabolizes T-2 to 3′OH-T-2 by the 3′-hydroxylation of isovaleryl groups. *In vitro* enzyme assays of recombinant CYP1A5 expressed in DH5α further confirm that CYP1A5 can convert T-2 into TC-1 (3′OH-T-2). Therefore, CYP1A5 is critical for the metabolism of trichothecene mycotoxin in chickens.

## 1. Introduction

T-2 toxin, one of the primary members of the type-A trichothecenes, which are naturally occurring contaminants of agricultural commodities, has been reported to be produced by a species of Fusarium, which are commonly found in various cereal crops and processed grains [1]. Products from livestock and poultry are the main food sources for humans. Because of the high potential to transmit T-2 toxin and its metabolites via the edible tissues of farm animals, T-2 toxin and its metabolites in these livestock and poultry products appear to represent an important potential danger to human health [2–4].

T-2 toxin was first isolated from the mold *Fusarium tricinctum* (*Fusarium sporotrichioides*) [5]. Over the past several decades, many metabolites have been characterized, including two important oxidation products, 3′-hydroxy-T-2 and 3′-hydroxy-HT-2 toxins, that were identified in lactating cows and in chicken excreta and tissues [6,7]. Subsequently, *in vitro* metabolism studies suggested that the hydroxylation of T-2 and HT-2 toxins could be accomplished by cytochrome P450 supplemented with an NADPH-generating system in the liver homogenates of mice, monkeys, pigs and rats [8,9].

Hydroxylation of the isovaleryl groups of T-2 and its metabolites is a major detoxification pathway. In pigs, some CYP3As have been reported to transform T-2 into its hydroxylation products, but until now, the specific CYP subfamilies in chickens that transform T-2 toxin into its hydroxylation products have not been reported [10,11]. Herein, we investigated which cytochrome P450 isoforms in chicken were involved in T-2 metabolism. Our results confirmed that chicken CYP1A5 plays an important role in hydroxylating T-2 toxin into 3′-OH-T-2.

## 2. Results and Discussion

### 2.1. Expression Changes of Major Cytochrome P450 in Response to T-2 Exposure

The major human CYP isoforms involved in drug metabolism are CYP3A, CYP2D6, CYP1A2, CYP2C, and CYP2E1 [12]. Sequence alignment has been performed by the BLAST architecture at the NCBI site. It is found in chicken that CYP1A4 (NP_990478.1) and CYP1A5 (NP_990477.1) are 57% and 63% identical in amino acid sequence to human CYP1A2, respectively. CYP2C45 (NP_001001752.1), CYP2C18 (NP_001001757.1) and CYP2H1 (NP_001001616.1) are 57%, 57% and 57% identical to human CYP2C9 (NP_000762.2), respectively. Chicken CYP3A37 (NP_001001751.1) and CYP3A80 (XP_414782.1) are 51% and 59% identical to human CYP3A4, respectively. In the CYP2D family, CYP2D49 (NP_001182486.1) has the highest identity (56%) to human CYP2D6 (NP_000097.3). CYP2C45 (NP_001001752.1), CYP2C18 (NP_001001757.1) and CYP2H1 (NP_001001616.1) are 53%, 51% and 52% identical to human CYP2E1 (NP_000764.1), respectively. Based on the sequence similarity, it is speculated that CYP1A4, CYP1A5, CYP2C45, CYP2C18, CYP2H1, CYP3A37, CYP3A80 and CYP2D49 may be the major CYP isoforms involved in drug metabolism in chicken.

**Figure 1:**
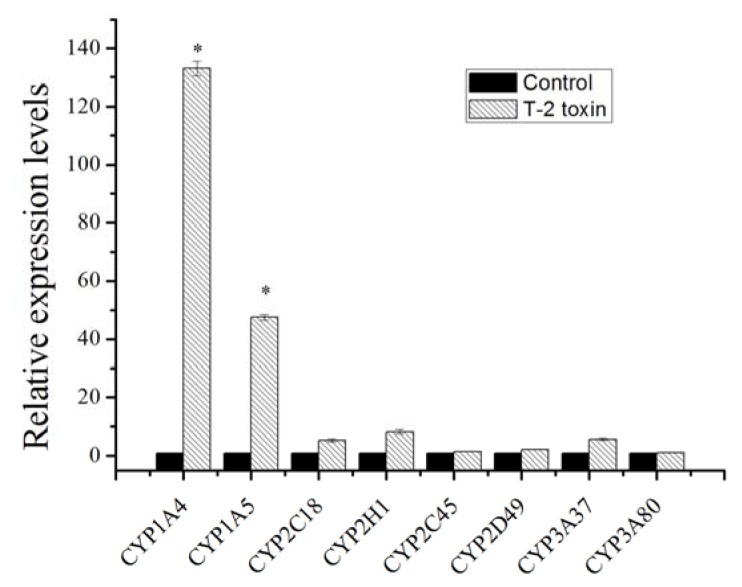
Quantitative real-time PCR of CYPs. Chicken embryonic hepatocyte cells were exposed to T-2 toxin at 0.1 μg/mL for 48 h. The mRNA levels of *CYP1A4* (Gene ID: 396052), *CYP1A5* (Gene ID: 396051), *CYP2C45* (Gene ID: 414833), *CYP2C18* (Gene ID: 414841), *CYP2H1* (Gene ID: 414746), *CYP3A37* (Gene ID: 414832), *CYP3A80* (Gene ID: 416477) and *CYP2D49* (Gene ID: 417981) were assessed by real-time PCR. The data are expressed as the mean ± SE of three independent determinations, and ANOVA was used for the statistical analysis. * *p* < 0.05, *n* = 3.

Therefore, the expression of these genes in chicken embryonic hepatocyte cells that were isolated after treatment with T-2 was investigated. The expression of *CYP1A4* and *CYP1A5* was substantially upregulated 132-fold and 47-fold, respectively (Figure 1). *CYP2C18*, *CYP2H1* and *CYP3A37* were induced 5.3-fold, 8.1-fold, and 5.7-fold, respectively. The other genes were not induced. Therefore, we speculated that CYP1A4 and CYP1A5 would be involved in the hydroxylation of T-2.

Mahajan and Rifkind reported that CYP1A5 was constitutively expressed in liver and kidney using more sensitive nuclear run on assays [13]. Gannon reported that 1A5 was induced by TCDD in kidney, as well as liver [14]. Liver is the major organ metabolizing exogenous and endogenous compounds.

In this paper, the magnitude of CYP1A4 response to 0.1 μg/mL T-2 is larger than that of others, but lacking the hydroxylation activity of T-2. The pattern of responsiveness is similar to previous research [14,15]. In their experiment, chicken embryo hepatocyte cultures exposed to 100 nM TCDD, CYP1A4 and CYP1A5 mRNA expressions were induced 61-fold and 25-fold, respectively. CYP1A5, but not CYP1A4, is an arachidonic acid epoxygenase.

In pigs, after T-2 toxin exposure, the mRNA levels of CYP1A2 were not significantly induced, but those of CYP3A22 and CYP3A46 were markedly induced [10,11]. Furthermore, *in vitro* catalysis assays suggested that the two CYP3As could metabolize T-2 to form 3′OH-T-2. In different species, the forms of P450 contributing to T-2 hydroxylation may be different. T-2 hydroxylation has been suggested to be performed by the sophisticated P450 enzyme system in animals, and other forms of P450 are also likely involved in this reaction in chickens, which requires further study.

### 2.2. The Catalytic Activity of S9 Fractions from HeLa-CYP1A4 and HeLa-CYP1A5

**Figure 2:**
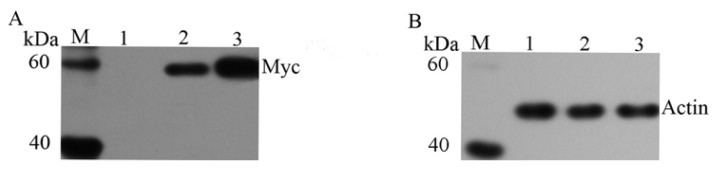
CYP1A4 and CYP1A5 expressed in HeLa cells. PcDNA-CYP1A4, pcDNA-CYP1A5 and empty vector were transformed into HeLa cells, and their transformants were confirmed by Western blotting using the myc-antibody (**A**) and actin-antibody (**B**). M: marker; 1: HeLa-pcDNA; 2: HeLa-CYP1A4; 3: HeLa-CYP1A5.

**Figure 3:**
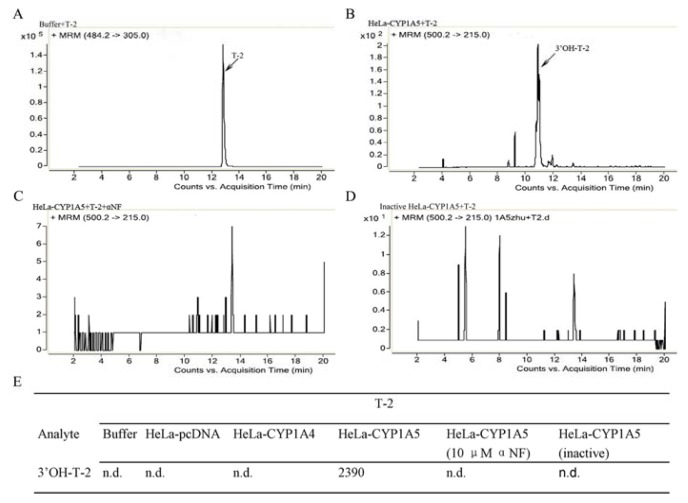
LC-ESI-MS/MS analysis of T-2 and its metabolites formed in HeLa transformants expressing CYP1A4 and CYP1A5. The detection was accomplished by MRM with the transitions *m*/*z* 484.2/305.0 for T-2 and *m*/*z* 500.2/215.0 for 3′-hydroxy-T-2. The data were presented as a spectrum (**A**–**D**) and as the values of the peak area (**E**). n.d., not detected.

CYP1A4 and CYP1A5-myc fusion proteins, each with an estimated molecular mass of 59 kDa, were detected with anti-myc antibodies, and β-actin antibodies were used as the control (Figure 2A,B). All these proteins were successfully expressed in HeLa cells. 7-Ethoxyresorufin, a human CYP1A subfamily substrate, was incubated with the S9 fractions. The HPLC assays suggested that the 7-ethoxyresorufin-O-deethylation was performed by the S9 fraction (from HeLa-CYP1A4 and HeLa-CYP1A5) to produce resorufin and that this reaction was effectively blocked by αNF (Figure S1A–D).The LC/MS experiments indicated that the S9 fractions from HeL-CYP1A5 generated 3′-hydroxy-T-2, but that the S9 fractions of HeLa-pcDNA and HeLa-CYP1A4 did not produce 3′-OH-T-2 (Figure 3). Interestingly, CYP2C18, 2H1 and 3A37 are also involved in the metabolism of T-2 toxin with different activities (data not shown).

In humans, CYP1A2 is a major hepatic P450 that plays a decisive role in the oxidation of many xenobiotic drugs and mycotoxins, such as caffeine, aflatoxin B1, polycyclic aromatic hydrocarbons (PAHs) and acetaminophen [16]. In this study, the mRNA levels of both CYP1A4 and CYP1A5 were upregulated many-fold, but only CYP1A5 could hydroxylate T-2 toxin. These findings are similar to those reported by Gannon *et al*. [14]. In their studies, both CYP1A4 and CYP1A5 in the chicken liver were induced by the environmental toxin, 2,3,7,8-tetrachlorodibenzo-*p*-dioxin (TCDD), but CYP1A5 possessed arachidonic acid epoxygenase activity. CYP1A5, similar to human CYP1A2, has been hypothesized to have an important role in the metabolism and detoxification of toxins. Chicken cytochrome P450 1A5 is the key enzyme for metabolizing T-2 Toxin to 3′OH-T-2. It has been shown in previous research that the mRNA of CYP1A4 and CYP1A5 were induced 61-fold and 25-fold, respectively, when chicken embryo hepatocyte cultures were exposed to 100 nM TCDD. Nevertheless, only CYP1A5 has the activity of arachidonic acid epoxygenase [14,15]. These findings suggested that CYP1A4 may be easily induced by drugs, but the activity of metabolizing drugs is low, which is similar to our data. The phylogenetic analysis of avian CYPs produced a tree topology consistent with the orthology of avian CYP1A5s with mammalian CYP1A2s and avian CYP1A4s with mammalian CYP1A1s [17]. The mammalian CYP1A2s are much more active in drug metabolism than CYP1A1s [12]. Sequence alignment showed that chicken CYP1A4 (NP_990478.1) is 79% identical to CYP1A5 (NP_990477.1) in amino acid sequence. The differences of responsiveness to T-2 toxin and catalytic activities between CYP1A4 and CYP1A5 are probably due to some different amino acids between them, which required further study.

### 2.3. The Catalytic Activity and Secondary Structure of Recombinant CYP1A5

**Figure 4:**
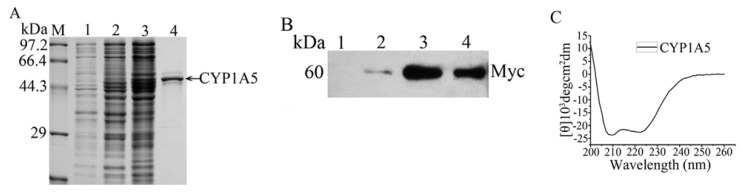
Expression, purification, secondary structure and Western blot detection of recombinant CYP1A5. The prokaryotic expression and purification of CYP1A5 were analyzed by 12% SDS-PAGE gels with Coomassie blue staining (**A**) and Western blotting using anti-myc antibodies (**B**). M: marker; 1: non-IPTG-induced; 2: IPTG-induced total cell lysates; 3: solubilized membrane fraction; 4: FPLC-purified CYP1A5. (**C**) The secondary structure of recombinant CYP1A5 was analyzed.

**Figure 5:**
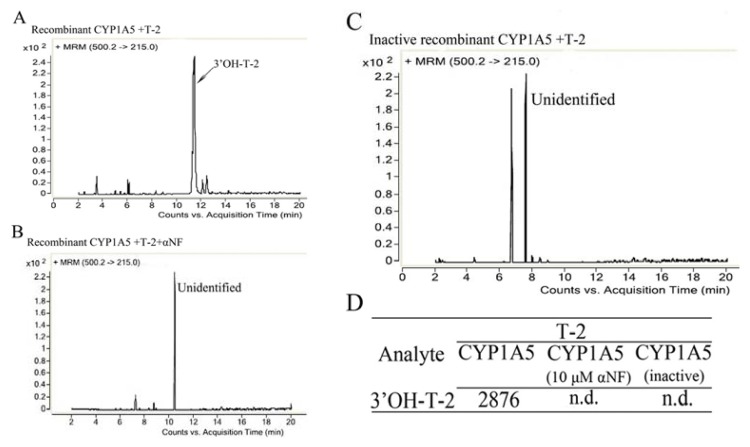
LC-MS/MS assays of T-2 after recombinant CYP1A5 biotransformation. T-2 was incubated with CYP1A5 (active or inactive) and in the reactions containing αNF or not. T-2 and its metabolites were detected and analyzed by LC-MS/MS. The data are represented as a spectrum (**A**–**C**) and as the values of the peak area (**D**). n.d., not detected.

To further confirm the metabolism of T-2 by CYP1A5 to 3′-OH-T-2, CYP1A5 was expressed in DH5α cells, purified and detected by SDS-PAGE and Western blotting with the anti-myc antibody (Figure 4A,B). The interaction of 7-ethoxyresorufin with purified CYP1A5 demonstrated that purified CYP1A5 could oxidize 7-ethoxyresorufin (Figure S1E,F). The incubation of T-2 with purified CYP1A5 also indicated that purified CYP1A5 interacted with T-2 to generate 3′-OH-T-2 (Figure 5A–D). According to the CATH classification, the structures of mammalian CYPs are mainly-helical [18,19]. Helical contents of human CYP1A2, CYP2C9 and CYP3A4 are 48%, 50% and 47%, respectively. Beta sheet contents of human CYP1A2, CYP2C9 and CYP3A4 are 9%, 9%, and 8%, respectively, from the web site (http://www.rcsb.org/pdb/home/home.do) [20–22]. Because of CYPs membrane-associated feature, we needed to make sure the recombinant CYP1A5 protein has the correct conformation, which is necessary to its activity. That’s why we examined the secondary structure of CYP1A5 to determine whether it correctly folded or not. The [θ] at 222 nm for CYP1A5 in Tris-HCl buffer (pH 7.4) was −22910 (equivalent to 69% helical content). The contents of the beta sheet, beta turn and random coil of chicken CYP1A5 are 6%, 11% and 14%, respectively, which has been analyzed by CDNN software, showing that purified CYP1A5 has a classic α-helical secondary structure (Figure 4C). Thus, recombinant CYP1A5 in Tris-HCl buffer is stable, because of the high helical content, which is beneficial to exert its catalytic activities.

## 3. Experimental Section

### 3.1. Chicken Embryonic Hepatocytes Cell Isolation, Culture and Exposure to T-2

Hepatocytes were isolated from the livers of 18-day-old chicken embryos by perfusion, and then, primary chicken embryonic hepatocytes were cultured [10]. After a 24 h growth period, the monolayer hepatocytes cultures were exposed to 0.1 μg/mL T-2 (dissolved in DMSO) or to an equal volume of DMSO used as a control. After a 48 h culture, samples were harvested to isolate RNA.

The 0.1 μg/mL T-2 dose was selected for treatment based on our previous MTT assay [10,11]. After exposure to different levels of T-2 toxin for 48 h, the IC_50_ of T-2 toxin for pig hepatocytes was determined to be 0.124 μg/mL. We also performed the MTT assay for chicken embryo hepatocytes exposed to different levels of T-2 toxin for 48 h. The IC_50_ of T-2 toxin for chicken embryo hepatocytes was determined to be 0.068 μg/mL. T-2 toxin is a naturally occurring contaminant of agricultural commodities. It has been shown that the average T-2 toxin contamination in chicken feed ranges between 0.03 and 0.155 mg/kg [23–26]. The concentration of T-2 toxin used (0.1 μg/mL) in this study is just located at this range.

### 3.2. RNA Isolation and Quantitative Real-Time PCR Analysis

Total RNA was extracted from the chicken embryonic hepatocytes, according to the manufacturer’s protocol using the TRIzol reagent method (Invitrogen, Carlsbad, CA, USA). Multiple genes (including *CYP1A4*, *CYP1A5*, *CYP2C18*, *CYP2H1*, *CYP2C45*, *CYP2D49*, *CYP3A37*, and *CYP3A80*) were amplified and analyzed by quantitative real-time PCR. Real-time PCR reactions using SYBR Green were performed with the Stratagene Mx3000P qPCR system (Stratagene, La Jolla, CA, USA). Specific primers were designed based on the real-time PCR experimental requirements. The melting curve analysis (60–95 °C) and gel electrophoresis (2% agarose) were used for assessing amplification specificity. PCR products were verified by sequencing.

The primers used are presented in Table S1. The data are reported as the mean ± SEM and were analyzed by ANOVA. *p* < 0.05 was considered to indicate a significant difference.

### 3.3. Vector Construction, Cell Culture, and Transfection and S9 Preparation

The open reading frame (ORF) regions of the chicken CYP1A4 and CYP1A5 genes were cloned into the *NotI*/*KpnI* sites of the pcDNA™3.1/myc-His(-)A vector (Invitrogen, Carlsbad, CA, USA) by PCR amplification and verified by the sequencing of cDNA samples from chicken embryonic hepatocytes. HeLa cell culture, transfection with these plasmids (pcDNA-CYP1A4, pcDNA-CYP1A5 and empty vector) and S9 fraction preparation and detection by SDS-PAGE and Western blotting were performed as described previously [27].

### 3.4. Enzyme Assay

T-2 toxin incubation with the S9 fractions from HeLa-CYP1A4 and HeLa-CYP1A5 and the detection of its metabolites were performed as described previously [11]. To study the metabolism of 7-ethoxyresorufin by S9 fractions, only the toxin was replaced by 7-ethoxyresorufin in the reaction system. The following modified incubation method was used to detect the catalytic activity of purified CYP1A5. The premix system contained 0.25 mg/mL dilauroylphosphatidyl choline, 0.2 μM NADPH-P450 reductase, 0.2 μM cytochrome B5, 0.2 μM recombinant CYP1A5 and 30 mM MgCl_2_. The reaction was initiated by the addition of 1 mM NADPH and T-2 and conducted at 37 °C with shaking for 3 h.

T-2 and the metabolites were identified by a LC-MS/MS instrument (1200RRLC-6410MS/MS) from Agilent Technologies (Waldbronn, Germany) equipped with an electrospray ionization (ESI) interface. A ZORBAX Eclipse Plus C18 column (100 mm × 2.1 mm, 1.8 μm) was used for chromatographic separation. In brief, the mobile phase A and B referred to water containing 5 mM of ammonium acetate and acetonitrile, respectively. The details of the gradient were depicted as follows: 0 to 5 min, 20% B; frequently from 20% to 65% B, 5 to 6 min and then, 65% to 80% B, 6 to 10 min; 80% B for 4 min; 80% to 20% B in 1 min and then, 20% B for 14 min. The flow rate was 0.2 mL/min; 2 μL of samples were injected onto the chromatographic column. For ESI, conditions were set as in our previous research [11].

To characterize the 7-Ethoxyresorufin *O*-deethylase (EROD) activity, in all reaction systems, only the toxin was replaced by 100 μM 7-ethoxyresorufin, and the interaction was conducted for 30 min. An equal volume of ice-cold acetone was added with vortexing to terminate the reaction, and the coagulated protein was precipitated by centrifugation at 5,000 rpm for 10 min. Then, 10 μL of the supernatant was injected into a Hypersil BDS C18 column (250 × 4.6 mm, 5 μM; Eliter, Dalian, China) and analyzed by HPLC (Waters Alliance, Milford, CT, USA) [28]. In all the control groups, the samples inactivated by heating were used, but in the inhibition experiments, the abovementioned pre-incubated mixture was supplemented with 10 μM α-naphthoflavone (αNF).

### 3.5. Recombinant Protein Expression and Purification in Prokaryotes and Circular Dichroism (CD) Spectroscopy Detection

For the functional expression in *E. coli*, a modification strategy (ompA + 2 strategy) was developed [29]. Recombinant CYP1A5 cDNA was digested using the *NotI* and *KpnI* sites present in the pcDNA-CYP1A5 plasmid and subsequently ligated into the pCWOri vector fragment of the pCWOri-CYP2D49 plasmid (constructed by our lab) [28] that was digested by *NotI* and *KpnI*, thereby replacing the CYP2D49 ORF. Recombinant CYP1A5 expression, membrane preparation and solubilization, purification and detection by SDS-PAGE and Western blotting were performed as previously described [27]. The CD spectra of the purified CYP1A5 were obtained on the Chirascan CD spectrometer (Applied Photophysics Limited, Leatherhead, UK) by the method previously described to detect the secondary structure [30].

## 4. Conclusions

In summary, the *in vitro* metabolism assays provide strong evidence that in the chicken, T-2 is first transformed into 3′-OH-T-2 by CYP1A5 through 3′-hydroxylation. Therefore, our research provides a strong theoretical support to understand not only the catalytic activity, regulation and detoxification role of CYP1A5, but also the mechanism of T-2 biotransformation in chickens.

## Supplementary Information
