# Supplementary material for: Chicken Cytochrome P450 1A5 Is the Key Enzyme for Metabolizing T-2 Toxin to 3′OH-T-2
Source: Int J Mol Sci. 2013 May 23;14(6):10809–18. doi: 10.3390/ijms140610809 (PMC3709703; doi:10.3390/ijms140610809)
Supplement: Supplementary file 1 [file ijms-14-10809-s001.pdf]

Supplementary Information

Figure S1. 7-Ethoxyresorufin *O*-deethylase (EROD) assay of recombinant CYP1A5 and S9 prepared from HeLa-CYP1A4 and HeLa-CYP1A5. 100 µM 7-ethoxyresorufin was incubated with 0.6 mg S9 prepared from HeLa-CYP1A4 and HeLa-CYP1A5, containing 10µM αNF or not. 7-ethoxyresorufin was metabolized by S9 prepared from HeLa-CYP1A4 without αNF (**A**), or with αNF (**B**). 7-ethoxyresorufin was metabolized by S9 prepared from HeLa-CYP1A5 without αNF (**C**), or with αNF (**D**). 7-Ethoxyresorufin *O*-deethylase (EROD) assay was performed with recombinant CYP1A5 in a reconstituted system and containing 10 µM αNF or not. The oxidative products of 7-Ethoxyresorufin were present at around 2.5 minutes (**E**). The metabolite peak was depressed significantly by the addition of αNF (**F**).

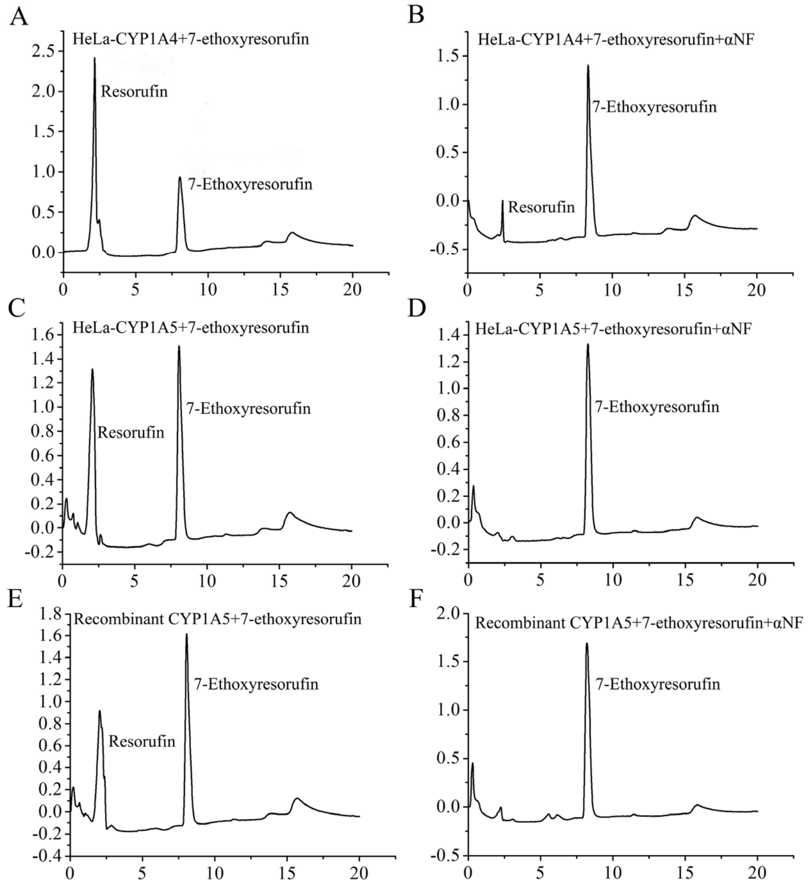

Table S1. Primers used for real-time PCR. Primers used to analyze mRNA level of chicken cytochrome P450s (including CYP1A4, CYP1A5, CYP2C18, CYP2C45, CYP2H1, CYP2D49, CYP3A37, and CYP3A80) after chicken embryos hepatocyte cells exposed to T-2 by real-time PCR.

Chicken Cytochrome P450	Primers for real-time PCR
CYP3A80 XM_003210584	Forward: CACCGTGACCCGGCGTACT<br>Reverse: TTCTGCGGATCACTGTGGG
CYP2 H1 NM_001001616.1	Forward: TGGGAGAGGAATACTGCCT<br>Reverse: TGGATTAAGAACTTCCCAGGG
CYP2C19 NM_001001757.1	Forward: GTGGGAGAGGCAATCTGC<br>Reverse: TTGAAAGGTTTCTCGTGTGTG
CYP1A5 NM_205146.2	Forward: GGACCGTTGCGTGTTTAT<br>Reverse: CTCCCACTTGCCTATGTTTT
CYP1A4 NM_205147.1	Forward: TCAATGCTCGTTTCAGTGCC<br>Reverse: AAGGCAGCGTACATCATGCA
CYP3A37 NM_001001751.2	Forward: TAAGGCTCCGCTCACGTA<br>Reverse: GGTGCAGGGTGTAAGGTG
CYP2D49 NM_001195557	Forward: GGCAAAGGGTAAGGAGGCT<br>Reverse: TGACGGCATTGGTGTAGGG
B-ACTIN NM_205518.1	Forward: GGCTGTGCTGTCCCTGTA<br>Reverse: CGGCTGTGGTGGTGAAG
CYP2C45 NM_001001752.1	Forward: GCTTGCCTGCTCTCCATC<br>Reverse: TCAAGGCTTCTTTCACCG

© 2013 by the authors; licensee MDPI, Basel, Switzerland. This article is an open access article distributed under the terms and conditions of the Creative Commons Attribution license (http://creativecommons.org/licenses/by/3.0/).
